# Early use of hemoadsorption in patients after out-of hospital cardiac arrest – a matched pair analysis

**DOI:** 10.1371/journal.pone.0241709

**Published:** 2020-11-03

**Authors:** Muharrem Akin, Vera Garcheva, Jan-Thorben Sieweke, Ulrike Flierl, Hannah C. Daum, Johann Bauersachs, Andreas Schäfer

**Affiliations:** Department of Cardiology and Angiology, Cardiac Arrest Centre, Hannover Medical School, Hannover, Germany; IRCCS Policlinico S.Donato, ITALY

## Abstract

**Background:**

Pro- and anti-inflammatory mediators are released during and after cardiac arrest, which may be unfavourable. Small case-series and observational studies suggested that unselective hemoadsorption may reduce inadequately high cytokine levels during sepsis or cardiac surgery. We aimed to assess the effect of cytokine adsorbtion on mortality in patients following out-of-hospital cardiac arrest by comparing a patient cohort with hemoadsorption after resuscitation for out-of-hospital cardiac arrest to a control cohort without adsorption within the HAnnover COling REgistry (HACORE).

**Methods:**

We adopted an early routine use of hemoadsorption in patients after out-of-hospital cardiac arrest with increased vasopressor need and performed a 1:2 match according to age, gender, time to return of spontaneous circulation, initial left-ventricular ejection fraction, extracorporeal membrane-oxygenation or left-ventricular unloading by Impella, need for renal replacement therapy, admission lactate, pH, glomerular filtration rate to patients without an adsorber from HACORE. The primary endpoint was 30-day mortality.

**Results:**

Twenty-four patients receiving hemoadsorption were matched to 48 patients without hemoadsorption (mean age 62±13 years, 83% male). While there was no significant difference in baseline parameters, 30-day mortality was higher in patients treated with hemoadsorption than in the matched control group (83% vs 65%, Log rank p = 0.011).

**Conclusions:**

Routine use of hemoadsorption did not reduce, but seems to be associated with higher 30-day mortality in patients after OHCA. Prior to routine adoption in daily practice, hemoadsorption should be evaluated in properly sized randomized controlled trials.

## Introduction

Ischemia and reperfusion injury during and after cardiac arrest triggers the release of many signaling molecules such as pro- and anti-inflammatory cytokines, tumor necrosis factors (TNF) and matrix metalloproteinases. As a result, post-cardiac arrest syndrome frequently occurs and is causally involved in brain injury, poor neurological outcome and myocardial dysfunction contributing to higher mortality after cardiac arrest [1]. Pro-inflammatory cytokines and soluble receptors are excessively released by leukocytes and endothelial cells following activation of metabolic cascades triggered by oxidative injury and further deteriorated hemodynamics, thus impairing end-organ function [2]. Elevated levels of Interleukin (IL)-6, IL-8 and TNF-α as well as of IL-1Ra and IL-10 have been observed in patients with poor neurological outcome and in non-survivors after cardiac arrest [3, 4]. However, as their mechanistic role and regulation after cardiac arrest is poorly understood, designation of these factors as pro- or anti-inflammatory is not possible in this condition. The beneficial effect of hypothermia as an important element in post-cardiac arrest care is attributed, among others, to suppression of cytokine release during the early phase after return of spontaneous circulation (ROSC) [1].

CytoSorb^®^ (CytoSorbents Corporation, Monmouth Junction, NJ, USA) is a polystyrene-based CE-certified device approved for conditions with elevated inflammatory mediators. CytoSorb^®^ adsorbs hydrophobic molecules with a size up to 55kD like interleukins (IL-6, IL-8, IL-10, IL-1β, C5a) and cytokines (TNF-α, IFN-γ, TNF-α monomer), damage-associated (CRP, HMGB-1, S-100) and pathogen-associated molecular patterns (streptococcus pyogenes exotoxin, clostridium perfringens toxin, staphylococcus aureus toxic shock toxin and haemolysin, aflatoxin) or metabolic products (bile acid, bilirubin, ammoniac) and other proteins (hemoglobin, ferritin, myoglobin) as well as drugs like antibiotics, platelet aggregation inhibitors, oral anticoagulants and others. Of note, the exact adsorption spectrum is not finally clarified [5, 6]. While observational studies, case series and expert recommendations suggest a clinical benefit of hemoadsorption in different conditions, larger randomized clinical trials to prove these hypotheses are lacking [5]. Positive effects of untargeted hemoadsorption in patients with sepsis or undergoing cardiac surgery are assumed. A significant decrease of IL-6 levels could be achieved in patients with sepsis, however, without a mortality benefit [7]. Other investigators could not confirm a significant decrease of IL-6 but of IL-8 levels [8]. CytoSorb^®^ use in cardiac surgery was associated with a decrease of cytokine levels in small case-control series [9, 10]. Apart from quantitative changes of cytokines in blood samples, there was no effect on mortality in these smaller cohorts.

Given the release of inflammatory mediators during post-cardiac arrest syndrome, hemoadsorption may potentially have a potential beneficial effect in out-of-hospital cardiac arrest (OHCA) patients with increased demand for vasopressors. Here, within the HAnnover COoling REgistry (HACORE), we compared the outcome of OHCA patients treated with hemoadsorption during a phase of routine application to controls without CytoSorb^®^, which had been treated according to the same post-arrest protocols [11].

## Methods

### Study ethics

HACORE is a prospective observational registry approved by the ethics committee at Hannover Medical School (#3567–2017) and is in accordance with the Declaration of Helsinki. The ethics committee approved the analysis as reported in the present manuscript. Written informed consent was obtained from legal guardians during the unconscious period and re-consented by survivors after gaining consciousness. HACORE includes anonymized data from all OHCA patients treated at our cardiac arrest centre with a standardized protocol including therapeutic hypothermia.

### Study design

We identified all patients from HACORE between 08/2017 and 08/2018 who underwent hemoadsorption with CytoSorb^®^ within 4 hours after intensive care unit (ICU) admission and performed a 1:2 match on known factors potentially contributing to mortality following successful resuscitation such as age, gender, time to ROSC, initial left ventricular ejection fraction, ECMO or Impella use, need for renal replacement therapy, admission lactate, pH, glomerular filtration rate (GFR) [12], to identify comparable patients without hemoadsorption from the time prior to routine adsorption (2012–2017). All patients had been admitted with OHCA to the cardiac arrest centre at Hannover Medical School and were treated according to the HaCRA-protocol as described before [11]. In brief all OHCA patients received a standard treatment by protocol including early determination of cardiac function and valvular disease by transthoracic echocardiography, diagnostic coronary angiography, computed tomography and therapeutic hypothermia after ICU admission for at least 24 hours at 32°C [11]. CytoSorb® was routinely applied during the investigational period when high-dose vasopressor demand defined as >0.3 μg/kg body weight/minute was detected in OHCA patients on ICU during the first 4 hours after cardiac arrest and used in addition to either renal replacement therapy, ECMO or both. The adsorber was installed preferentially within the dialysis circuit. There was no patient in whom extracorporeal circulation was established solely for CytoSorb^®^ application. Use of CytoSorb^®^ was attempted for three days.

High-dose vasopressor use is associated with higher probability for mortality in patients with cardiogenic shock [13, 14]. Maximal dosage of vasopressors expressed in μg / kg /minute were calculated during the first 4 hours for the control group and prior to CytoSorb^®^ application for the treated group.

Until the end of the period when hemoadsorption was considered in patients receiving standardized care following OHCA, 430 patients had been enrolled in HACORE, of which 158 patients had higher vasopressor demand (27 were treated with CytoSorb^®^, 131 had been treated prior to that period and served as potential matching controls). Three patients were excluded from further analysis due to missing matching partners. Finally, 24 eligible patients were matched in a 1:2 fashion to 48 patients without CytoSorb^®^.

Primary endpoint was 30 day mortality, secondary endpoint was neurological outcome at final hospital discharge. Neurological status was assessed by cerebral performance category.

### Clinical follow-up

Patients were followed up for the period of their hospital stay and data were extracted from the electronic hospital patient data management system. Discharge letters from rehabilitation facilities were collected when patients had been transferred to such an institution.

### Statistical analysis

Baseline characteristics are presented as frequencies (n) and percentages (%) for categorical variables, means ± standard deviation (SD) for normally distributed continuous variables, or median and interquartile ranges (IQR) for non-normally distributed continuous variables. Normally distributed variables were compared by Student´s t-test and Mann-Whitney test for nonparametric data, respectively. All group comparisons of continuous measures were performed using Wilcoxon’s test, whereas chi-square or Fisher’s exact test were used to assess categorical data. To control effects of potential confounders individual 1:2 case-control match was performed followed by comparison for non-significance as described above for categorical and continuous variables. Cumulative mortality was estimated by Kaplan-Meier method with statistical significance examined by the log-rank test. Statistical analyses were performed using SPSS Statistics 24 (IBM SPSS Statistics 24). A two-sided p-value of <0.05 was considered statistically significant.

## Results

During the period when hemoadsorption was considered in patients receiving standardized care following OHCA having higher vasopressor demand 24 eligible patients were matched in a 1:2 fashion to patients without CytoSorb^®^. All patients were initially in shock requiring high doses of volume administration and catecholamines. Baseline characteristics including matching criteria are shown in Table 1. There were no acute adverse effects related to the application of the device. CytoSorb^®^ was used either in addition to extracorporeal renal replacement therapy (n = 14; 58%), ECMO (n = 4; 17%) or both (n = 6; 25%).

**Table 1:** Baseline characteristics of matched HACORE patients with and without CytoSorb^®^ adsorber.

	CytoSorb group		matched group		p value
	(n = 24)		(n = 48)		
Age (years), mean±SD	62±13		61±13		0.898
Gender (male) (%)	20	(83)	40	(83)	1.000
ROSC (min), mean±SD	45±33		35±23		0.211
LV-EF:					0.944
normal (%)	4	(17)	9	(19)	
moderate (%)	7	(29)	15	(31)	
severe (%)	13	(54)	24	(50)	
ECMO (%)	8	(33)	17	(35)	0.863
Impella (%)	10	(42)	23	(48)	0.622
RRT on ICU (%)	20	(83)	40	(83)	1.000
Vasopressor demand μg/kg/min	0.63±0.26		0.56±0.36		0.530
**Admission laboratory**					
Lactate (mmol/l), mean±SD	11.0±4.2		10.3±4.5		0.476
pH, mean±SD	6.99±0.12		7.06±0.21		0.146
Creatinin (μmol/l), mean±SD	131±55		125±75		0.728
GFR (ml/min), mean±SD	60±27		64±25		0.536
CRP (μg/l), mean±SD	14.1±33.6		39.1±64.4		0.079
PCT (mg/l), mean±SD	1.3±2.2		4.3±11.2		0.191
WBC (1000/μl), mean±SD	17.9±8.2		14.5±6.9		0.093
**Comorbidities**					
Smoking (%)	5	(21)	17	(35)	0.205
Arterial hypertension (%)	10	(42)	28	(58)	0.182
Hyperlipidemia (%)	4	(17)	16	(33)	0.137
Family history for CAD (%)	2	(8)	1	(2)	0.211
Diabetes (%)	4	(17)	12	(25)	0.423
CAD (%)	5	(21)	8	(17)	0.665
Prior CABG (%)	1	(4)	4	(8)	0.512
Prior PCI (%)	1	(4)	2	(4)	1.000
PAD (%)	3	(13)	6	(13)	1.000
Stroke (%)	1	(4)	6	(13)	0.261
COPD (%)	2	(8)	5	(10)	0.778
Terminal kidney disease (%)	0	(0)	0	(0)	1.000
Atrial fibrillation (%)	4	(17)	8	(17)	1.000
Prior pacemaker (%)	2	(8)	1	(2)	0.211
Prior ICD (%)	0	(0)	1	(2)	0.476
**Arrest setting:**					0.866
private (%)	14	(58)	27	(56)	
public (%)	10	(42)	21	(44)	
Wittenessed arrest (%)	20	(83)	38	(79)	0.674
Bystander CPR (%)	17	(71)	28	(58)	0.302
Shockable primary rhythm (%)	13	(54)	32	(67)	0.302
Out of hospital defibrillations (IQR)	2 [0–6]		3 [1–5]		<0.001
Ongoing CPR (%)	8	(33)	13	(27)	0.582
**Cause of resuscitation:**					0.353
unknown (%)	5	(21)	5	(10)	
myocardial (%)	10	(42)	28	(58)	
arrhythmia (%)	5	(21)	11	(23)	
hypoxic (%)	3	(13)	4	(8)	
PE (%)	1	(4)	0	(0)	
PCI (%)	14	(50)	30	(63)	0.737
NSE (μg/l)^†^ (IQR)	47 [37–114]		48 [33–178]		0.888
S-100b (μg/l)^†^ (IQR)	0.178 [0.124–0.454]		0.163 [0.107–0.975]		0.587

BMI–body-mass-index; BP syst.–systolic blood pressure at admission; CAD–coronary artery disease; CABG–coronary artery bypass graft; COPD–chronic obstructive pulmonary disease; CPR- cardiopulmonary resuscitation; CRP–c- reactive protein; ECMO–extracorporeal membrane oxygenation; GFR–glomerular filtration rate; HACORE–HAnnover COoling REgistry; ICD–intracardial defibrillator; LV-EF–left ventricular ejection fraction; NSE–neuron specific enolase; PE–pulmonary embolism; PAD–peripheral artery disease; PCI–percutaneous coronary intervention; PCT–procalcitonine; ROSC–return of spontaneous circulation; RRT–renal replacement therapy; WBC–white blood cells.

^†^NSE and S-100 were determined on day 3 after admission.

As expected, there was no significant difference for matching factors like age, gender, time to ROSC, initial left-ventricular systolic function, use of ECMO or Impella, admission lactate, pH, creatinine, or glomerular filtration rate (Table 1). Moreover, cardiovascular risk factors such as smoking, arterial hypertension, hyperlipidemia, family history for coronary artery disease or diabetes were similar among these groups. There was no significant difference according to previous morbidities like obstructive pulmonary diseases, stroke and previous coronary interventions (Table 1). Importantly, preclinical factors such as cause of resuscitation, witnessed arrest, bystander cardiopulmonary resuscitation (CPR), ongoing CPR or PCI rate were comparable between CytoSorb^®^ and control groups. There was, however, a significant difference regarding the number of preclinically applied defibrillations per patient, whereas the prevalence of shockable primary rhythms was not significantly different (Table 1).

Within the first 24 hours, there was no difference between the two groups in terms of volume requirement or inotropic demand. There was no significant difference for potential prognostically relevant factors such as NSE and S-100b after OHCA even though mean biomarker levels were elevated and, therefore, suspicious for unfavorable outcome in both groups (Table 1). As these biomarkers were obtained on day 3 by protocol, they are only eligible for patients who had survived for at least 3 days.

**Fig 1:**
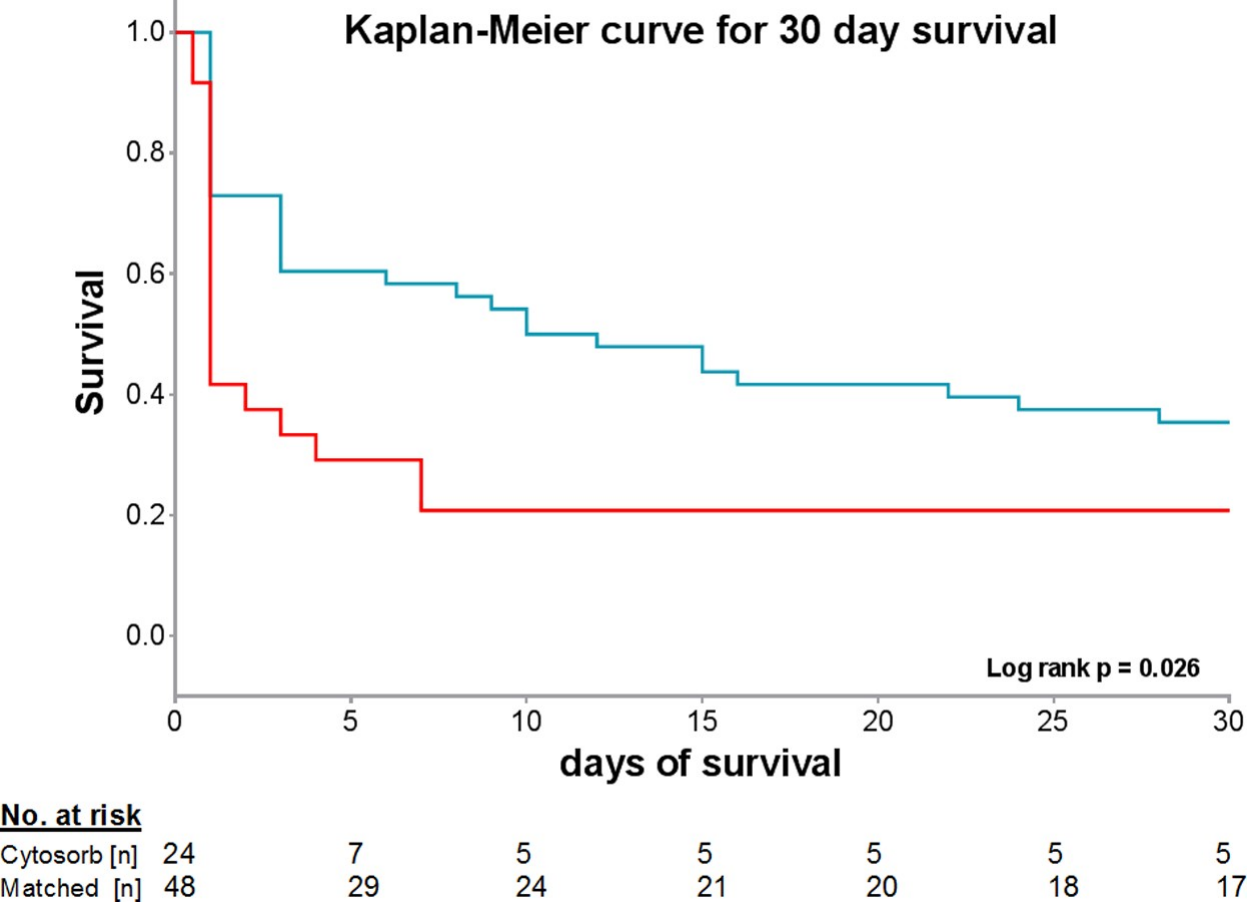
Kaplan-Meier curve for 30-day mortality in the CytoSorb^®^ (red line) and matched control group (blue line).

**Fig 2:**
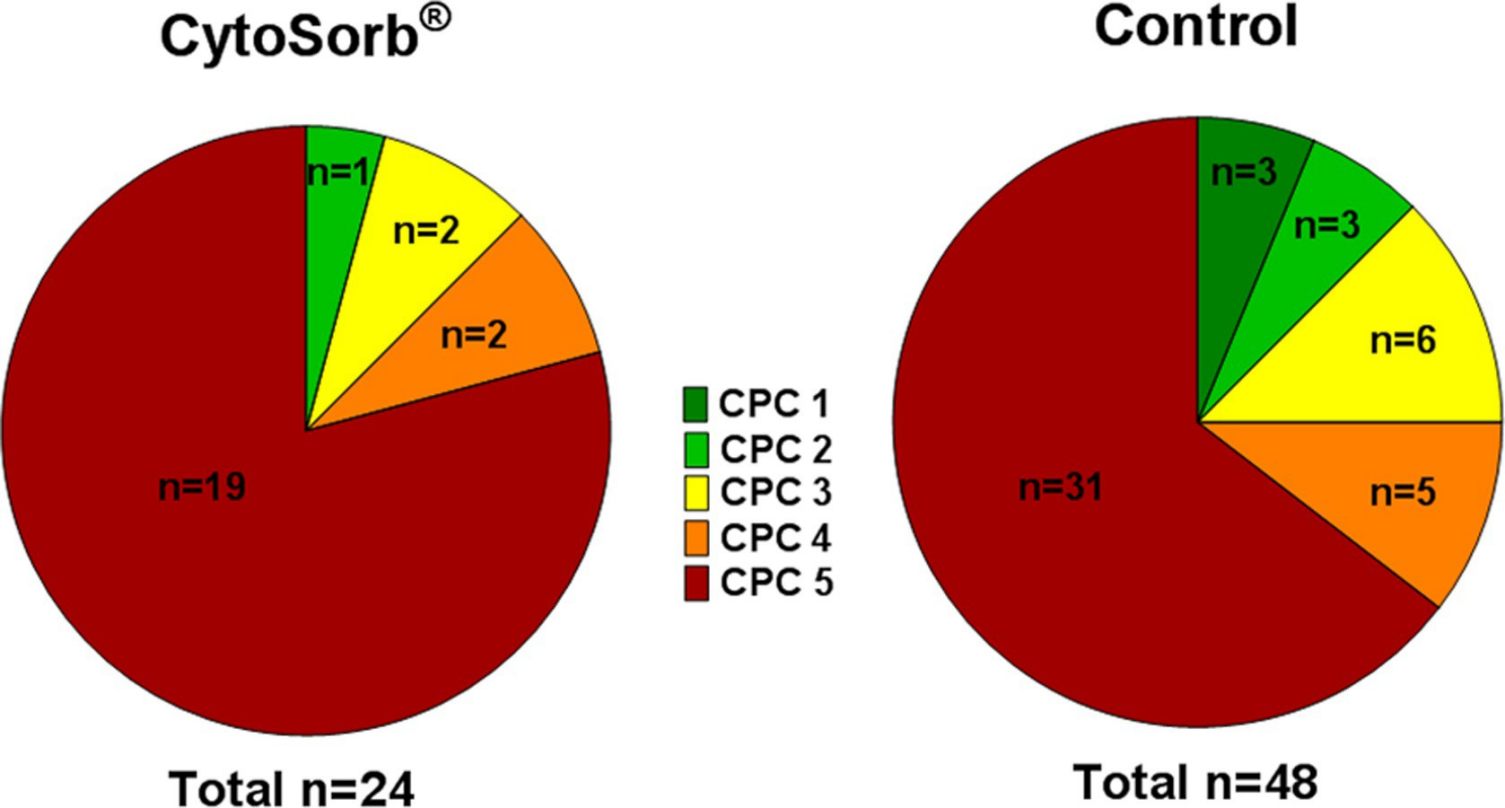
30-day-neurological outcome of survivors in the CytoSorb^®^ and matched control group.

Despite similar hemodynamic and intensive care characteristics among both groups, however, 30-day mortality was significantly higher in CytoSorb^®^ treated patients than in the control group (p = 0.026, Fig 1). Survivors in the matched control-group had a more favorable neurological outcome according to CPC-category compared to those surviving on CyotSorb^®^ (Fig 2). However, despite that our findings are observational and from a rather small matched cohort.

## Discussion

In this observational comparison of OHCA patients with increased vasopressor demand routinely treated with hemoadsorption using CytoSorb^®^, we could not find a clinical benefit regarding mortality. In contrast, in this non-randomized analysis we observed an increased 30-day mortality (83% vs. 64%; Log rank p = 0.011) in patients who underwent hemoadsorption. Survivors in the control group showed numerically more often a good neurological outcome (CPC ≤2) than patients in the CytoSorb^®^ group (35% vs. 20%; p = 0.47).

Although our rather small cohort lacked the necessary power to draw definitive conclusions, we did not continue routine application of CytoSorb^®^ in post-arrest patients with increased vasopressor demand as we considered it to be unethical to prolong our observation without data from a properly sized, randomized controlled trial. The absolute mortality difference of 19% based on the observed mortality rates of 83% in the CytoSorb^®^ group and 64% in the matched control group would require a total of 192 patients in a randomized controlled setting to reliably state with 80% power and a two-sided alpha error of 5% that such an effect is indeed significant.

CytoSorb^®^ has mostly been investigated in patients with sepsis or cardiac surgery on cardiopulmonary bypass, and its use is widely based on reported experience in case series or expert recommendations. In sepsis, organ dysfunction caused by a dysregulated host response induced by pathogen-associated molecule patterns via direct cellular damage or excessive cytokine production can lead to a systemic inflammatory response syndrome and thereby to life-threatening organ dysfunction. In vitro a significant reduction for IL-6, IFN-y, MIP-1α, C5a, HMGB-1, procalcitonin and S100-A8, but not of TNF-α could be achieved in blood samples with sepsis-like high cytokine levels by binding to CytoSorb^®^ hemoadsorbent polystyrene beads [15]. It was assumed that this effect could mitigate an excessive and undirected inflammatory response and, therefore, might have a positive outcome effect by suppressing an excessive inflammatory reaction. However, the immune response and inflammatory reaction in sepsis are not universally comparable to the biological mechanisms involved after OHCA, and therefore assumptions and observations in sepsis and other conditions might not be translated to OHCA patients.

In a small prospective, underpowered, nonrandomized single-center study using CytoSorb^®^ as a rescue treatment in 20 patients with refractory septic shock, beneficial effects on vasopressor need and decreased interleukin and lactate concentrations have been observed [16]. Mortality was lower than predicted based on the SOFA score, in the absence of direct or indirect comparisons groups, and the authors concluded on a beneficial effect of CytoSorb^®^ on mortality. However, there was neither a control group nor a historic control group treated by the same protocol. In another propensity score weighted retrospective study using CytoSorb^®^ in patients with septic shock, observed mortality was lower expected in patients with than in those without use of CytoSorb^®^ [17]. However, factors like older age and pneumogenic sepsis as origin of sepsis were more frequent in the control group.

Overall, there are no randomized controlled trials demonstrating a mortality benefit for unselected adsorption of hydrophobic substances using CytoSorb^®^. CytoSorb^®^ adsorbs both pro- and anti-inflammatory mediators, and the interference with certain medications given during post-cardiac arrest care remains unclear. Although several limitations apply, we observed a higher mortality in a cohort receiving hemoadsorption with CytoSorb^®^, thus safety and efficacy of this treatment in patients after OHCA remain unclear. Notwithstanding that interventions to control excessive ischemia-reperfusion syndrome after OHCA are urgently needed. Therefore, a prospective and appropriately sized randomized controlled trial in patients following OHCA is warranted before the device is recommended for routine use in this condition in the absence of safety data.

### Limitations

The number of patients examined was quite small. Therefore, larger studies with clear inclusion criteria for use of CytoSorb^®^ are required.

Despite lack of relevant differences in baseline characteristics, we cannot rule out that patients in the CytoSorb^®^ group might have been sicker, which may contribute to a higher mortality rate. Some indicators for worse outcome were more frequent in the CytoSorb^®^ group (time to ROSC was longer, less patients had shockable rhythms and primary PCI) but did not significantly differ to controls. On the other hand some parameters predisposing to better outcome were more common in the CytosSorb^®^ group (lower CRP and PCT levels; more frequent witnessed arrest and bystander CPR).

While our results do not provide evidence for increased mortality by CytoSorb^®^ use, nevertheless the present finding represents a potential safety concern, which should be addressed in randomized, controlled trial prior to routine implementation in daily clinical practice.

## Conclusion

Hemadsorption could not reduce 30-day mortality in critically ill patients after out of hospital cardiac arrest and seems to be associated with higher 30-day mortality. Therefore prior to adoption in daily practice, effects on outcome should be investigated in properly sized randomized controlled trials.
